# Supramolecular One-Dimensional n/p-Nanofibers

**DOI:** 10.1038/srep14154

**Published:** 2015-09-15

**Authors:** Alberto Insuasty, Carmen Atienza, Juan Luis López, Juan Marco-Martínez, Santiago Casado, Avishek Saha, Dirk M. Guldi, Nazario Martín

**Affiliations:** 1 Departamento de Química Orgánica I, Facultad de Ciencias Químicas, Universidad Complutense de Madrid, E-28040 Madrid, Spain; 2 IMDEA- Nanoscience, Campus de Cantoblanco, E-28049 Madrid, Spain; 3 Department of Chemistry and Pharmacy & Interdisciplinary Center for Molecular Materials, Friedrich-Alexander-University Erlangen-Nuremberg, 91058 Erlangen, Germany

## Abstract

Currently, there is a broad interest in the control over creating ordered electroactive nanostructures, in which electron donors and acceptors are organized at similar length scales. In this article, a simple and efficient procedure is reported en-route towards the construction of 1D arrays of crystalline pristine C_60_ and phenyl-C_61_-butyric acid methyl ester (PCBM) coated onto supramolecular fibers based on exTTF-pentapeptides. The resulting n/p-nanohybrids have been fully characterized by a variety of spectroscopic (FTIR, UV-Vis, circular dichroism, Raman and transient absorption), microscopic (AFM, TEM, and SEM), and powder diffraction (X-ray) techniques. Our experimental findings document the tendency of electroactive exTTF-fibers to induce the crystallization of C_60_ and PCBM, on one hand, and to afford 1D n/p-nanohybrids, on the other hand. Furthermore, photogenerated radical ion pairs, formed upon visible light irradiation of the n/p-nanohybrids, feature lifetimes on the range of 0.9–1.2 ns.

Owing to their remarkable electronic, mechanical and geometrical properties, C_60_ and its derivatives have drawn much attention during recent decades^1^,^2^,^3^. A plethora of reported C_60_ derivatives has made its way into emerging electronic applications such as photovoltaic cells^4^,^5^,^6^,^7^,^8^,^9^, organic transistors^10^,^11^,^12^,^13^,^14^,^15^,^16^, organic light emitting diodes (OLEDs)^17^,^18^,^19^,^20^,^21^,^22^, and sensors^23^,^24^,^25^,^26^,^27^,^28^,^29^. Importantly, the mutual dependence between morphology/crystallinity of the photoactive layers and device efficiencies is well established. For example, in bulk heterojunction (BHJ) photovoltaic devices, the existence of nanostructured domains, in which electron donors and/or electron acceptors are ordered at the same length scale, is of key importance to boost the overall efficiency^30^,^31^. Several molecular building blocks, which are integrated into photoactive layers, do not lead to the formation of nanostructures and/or microstructures that fulfill the aforementioned requirements. Still, the use of additives, as a means to control the nanostructure and/or microstructure in photoactive layers, constitutes a powerful approach. In particular, the employment of nucleating agents exerts a strong impact on the charge mobility in the resulting films^32^.

In recent years, C_60_ derivatives featuring an amphiphilic character have evolved as good candidates for being integrated into active layers yielding hierarchical architectures across different length scales^33^,^34^,^35^,^36^,^37^. Notably, several major drawbacks remain. Among them is the need for tedious synthetic protocols to prepare amphiphilic C_60_ derivatives as well as the challenge to optimize conditions for the efficient intermolecular assembly between the different building blocks. Frequently, the self-organization of amphiphilic C_60_ derivatives onto nanostructures and/or microstructures renders rather hard and, typically, leads to amorphous materials. Therefore, developing simple procedures to generate 1D nanostructures, but circumventing any of the aforementioned drawbacks, constitutes a contemporary challenge.

Inspired by biomineralization^38^,^39^,^40^,^41^,^42^,^43^,^44^, we have devised a simple and general strategy towards 1D n/p-nanostructures^45^,^46^,^47^,^48^,^49^ based on coating exTTF-based peptides fibers with electron accepting C_60_. We have focused on pristine C_60_ and phenyl-C_61_-butyric acid methyl ester (PCBM), as an n-type organic building block. Considering biomineralization, where peptide nanofibers govern the crystallization of inorganic crystals, we explored a similar role for exTTF-fibers. To this end, the latter serves as a template to guide the growth of C_60_ and PCBM over the exTTF-fibers. Incentivies for this work are based on the potential use of exTTF-fibers as seed to induce the growth of ordered C_60_ on their surfaces to achieve, ultimately, 1D n/p-nanostructures. To the best of our knowledge, this approach is currently largely unexplored.

## Results and Discussion

**Figure 1:**
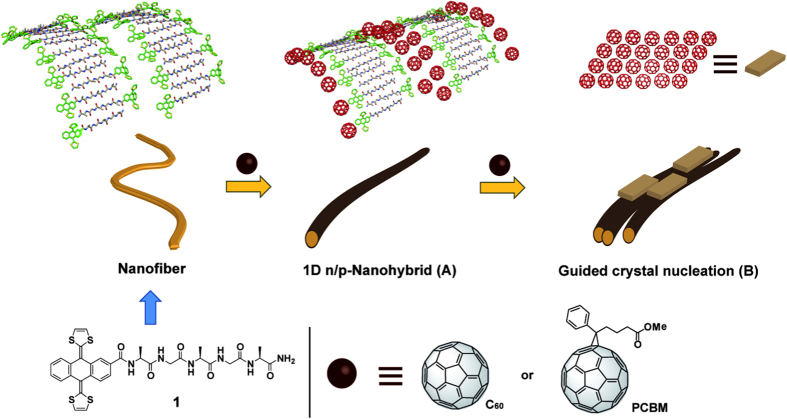
Chemical structures and cartoon showing the coating process of the exTTF fibers by C_60_ and PCBM leading to the formation of 1D n/p nanohybrids (A). The existence of C_60_ domains in the 1D n/p-nanohybrids triggers the oriented growth of crystal nuclei of C_60_/PCBM on top of them (**B**).

We have used well-known exTTF-fibers (**1**)^50^, where exTTF is covalently linked to a pentapeptide (Ala-Gly-Ala-Gly-Ala-NH_2_) in combination with pristine C_60_ as well as PCBM (Fig. 1) to explore the use of exTTF-fibers for the growth of C_60_/PCBM in the formation of 1D n/p-nanohybrids. Initially, we fabricated exTTF-fibers in 1,1,2,2-tetrachloroethane (TCE) followed by the addition of C_60_/PCBM (For more details about the formation of the exTTF-fibers see reference 50 and general methods). In this approach, two key effects are operative simultaneously. On one hand, strong non-covalent π-π interactions between C_60_ and exTTF promote the formation of 1D n/p-nanohybrids in which pristine C_60_/PCBM are assembled within exTTF-fiber’s nanostructure (see n/p-nanohybrid in Fig. 1A)^51^,^52^,^53^,^54^,^55^,^56^,^57^. On the other hand, the susceptibility of C_60_/PCBM to aggregate favours the cristalization over the nanohybrids (Fig. 1B). We reasoned that the combination of the earlier and the latter ensures an efficient interfacing between C_60_/PCBM and exTTF-fibers as a means of facilitating the growth of 1D n/p-nanohybrids. Our approach involves neither covalent chemical modifications nor optimization of the supramolecular self-assembly. We will demonstrate that a local supramolecular structure is achieved (Fig. 1A) and that nucleation of C_60_/PCBM crystals on the surface of nanohybrids (Fig. 1B) is followed. Analogous to peptide mineralization, the aforementioned accounts for the efficient interfacing between the different building blocks, that is, C_60_/PCBM and exTTF-fibers. To the best of our knowledge, the use of supramolecular nanostructures as seeds for ordering “electroactive molecules” such as C_60_/PCBM at the nanoscale is unprecedented.

**Figure 2:**
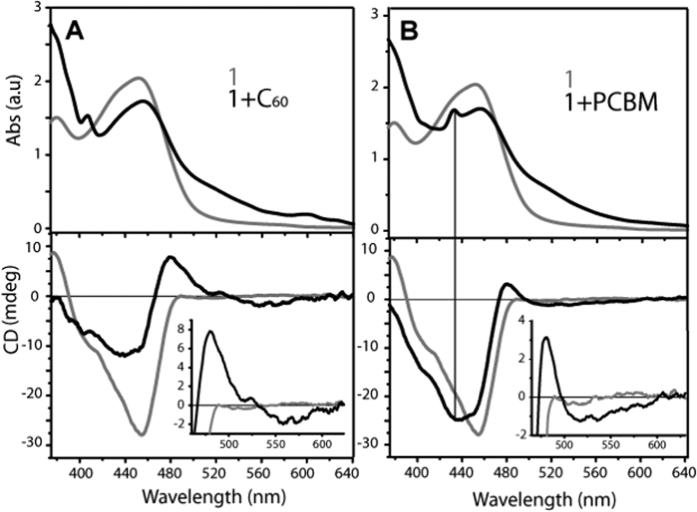
Ultraviolet-visible (UV-Vis) and circular dichroism (CD) spectra for exTTF-fiber and n/p-nanohybrids. (**A**) UV-Vis (upper part) and CD (lower part) spectra of **1** (grey line, 1.3 × 10^−3^ M) and the mixture of **1**:C_60_ (black line, 1.3 × 10^−3^ M) in TCE following aging for 5 days. (**B**) UV-Vis (upper part) and CD (lower part) spectra of **1** (grey line, 1.3 × 10^−3^ M) and **1**:PCBM (black line, 1.3 × 10^−3^ M) in TCE following aging for 5 days.

We have recently reported that exTTF-pentapeptide (**1**) in TCE solutions form complex networks of curved rope-like helical fibers with diameters ranging from 2 to 10 nm. Their overall organization is driven exclusively by H-bonds (*β*-sheets) without π-π interactions between exTTFs^50^. Room temperature absorption assays, which were carried out with **1** in TCE in the presence of either pristine C_60_/PCBM upon 5 days of aging, gave rise to a depletion and a red-shifting of the initial exTTF-fiber absorptions from 449 to 456 nm together with an increase in the absorption in the region >470 nm. These features are tentatively assigned to a reflection of π-π interactions between exTTF and C_60_^51^,^52^,^53^,^54^. Results obtained with C_60_/PCBM are shown in Fig. 2.

More pronounced were the changes noted in circular dichroism (CD) assays, in which the presence of C_60_ resulted in a positive Cotton effect at 483 nm and a negative Cotton effect at 439 nm (Fig. 2). In line with previous reports, the signal at 483 nm is attributed to π-π interacting C_60_ and exTTF in the new supramolecular helical arrangements^51^,^52^,^53^,^54^. An additional Cotton effect is seen in the 500–600 nm region, which is due to the fact that C_60_ is placed in a chiral environment owing to the lack of absorbance of the exTTF-fibers in this spectral region (Fig. 2A, inset). Similarly, CD experiments with PCBM showed a positive Cotton effect at 480 nm and a negative Cotton effect at 432 nm, which matches the typical absorptions of C_60_ monoadducts (Fig. 2B). Likewise, the new broad negative Cotton effect in the 500–600 nm region is also observed (Fig. 2B, inset). In conclusion, our findings attest the formation of chiral n/p-nanohybrids formed from exTTF-fibers and C_60_/PCBM. Hereby, the organization of exTTFs within the resulting n/p-nanohybrids has enabled the integration of C_60_/PCBM inside the exTTF-fibers leading to an overall new helical arrangements.

As a complement, the formation of the n/p-nanohybrids was followed by Raman experiments with C_60_/PCBM as references and the respective n/p-nanohybrids formed from mixtures of **1** and C_60_/PCBM. For C_60_, the characteristic *A*_*g*_*(2)* pentagonal pinch mode was observed at 1466 cm^−1^^36^. In stark contrast, the *A*_*g*_*(2)* mode gave rise in the n/p-nanohybrid to a slight shift towards lower frequencies, that is, 1461 cm^−1^. As such, interactions between exTTF-fibers and C_60_ are inferred. Similar trends were noticed for PCBM (Supplementary Fig. 1).

**Figure 3:**
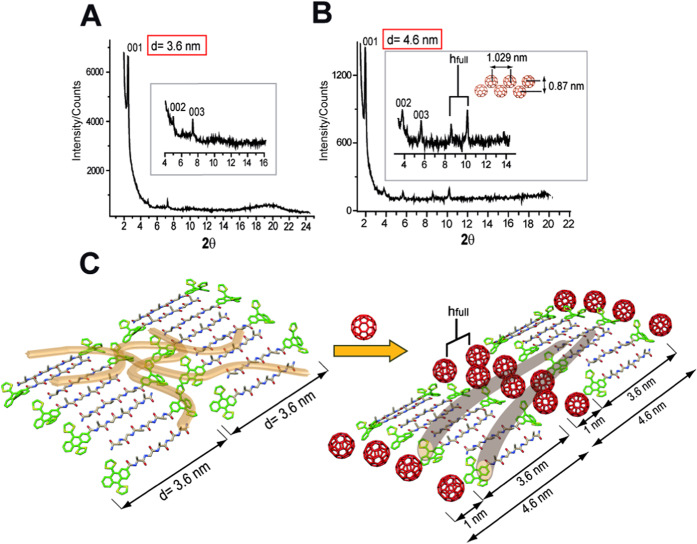
Powder X-ray diffraction (PXRD) for exTTF-fibers, n/p-nanohybrids and schematic illustration of the molecular packing for n/p-nanohybrids. (**A**) PXRD pattern for **1** obtained from a dispersion of exTTF-fibers in a mixture TCE:toluene. (**B**) PXRD pattern for **1**:C_60_ nanohybrid. In the inset, “h_full_” denotes the observed C_60_ reflections in the 2θ region between 8 and 11. (**C**) Cartoon showing the molecular packing observed for the exTTF-fibers and for the **1**:C_60_ nanohybrid. The distances observed by PXRD experiments are remarked in the cartoon.

Powder X-ray diffraction (PXRD) shed further light onto changes in the internal structure of the exTTF-fibers when they are tightly interfaced with C_60_/PCBM. From PXRD with **1** and C_60_ a new set of long range order with *d*-spacings at 4.6, 2.3 and 1.54 nm was derived (Fig. 3B). Such a new set is associated with lamellar packing originated from exTTF-fibers within the newly formed n/p-nanohybrids. The lamellar pattern with a *d*-spacing of 4.6 nm relative to 3.6 nm for exTTF-fibers infers an increase of about 1 nm which is attributed to the insertion of C_60_ within the nanostructure as shown in Fig. 3A–B. Furthermore, two signals with *d*-spacings of 1.02 and 0.87 nm were observed (denoted as h_full_, Fig. 3B, inset), which could be assigned to close packed columns of C_60_ intercalated between exTTFs (Fig. 3C)^58^,^59^,^60^,^61^,^62^,^63^,^64^. In the case of **1**:PCBM, despite PXRD did not show a clear pattern as **1**:C_60_, a broad signal with *d*-spacing of about 1 nm supports the notion of close packed columns of PCBMs (Supplementary Fig. 2). Please note that such a lamellar packing resembles that seen for blends based on semicrystalline polymers and C_60_ derivatives^65^.

Additionally, as a complement to support the proposed model for the n/p-nanohybrids (Fig. 3C), Fourier transform infrared experiments (FTIR) were carried out to confirm that intermolecular β-sheets in the peptide backbone are retained within the internal structure of the nanohybrids. The results in both cases (**1**:C_60_/PCBM), showed an intense amide I peak at around 1627 cm^−1^ together with a weak shoulder at around 1680 cm^−1^ thereby confirming the existence of β-sheets in an antiparallel mode (Supplementary Fig. 3).

The high tendency of C_60_ to aggregate by means of van der Waals interactions is likely to favor the evolution from an initial state, in which close packed columns of C_60_ are intercalated between exTTF-fibers (Fig. 1A and Fig. 3C), into a final state, where the C_60_ crystalize onto n/p-nanhybrids (Fig. 1B). Implicit are 1D n/p-nanohybrids, in which donor and acceptor units are organized at the same length scale (Fig. 1 and Fig. 3C)^46^,^47^,^48^,^49^,^50^,^51^,^52^. It opens a promising avenue to guide the crystallization of C_60_ on top of exTTF-fibers and/or exTTF-gels to afford anisotropic C_60_ crystals. This comes, however, without the needs of using C_60_ derivatives covalently linked to functional substituents that would facilitate such growth^61^.

**Figure 4:**
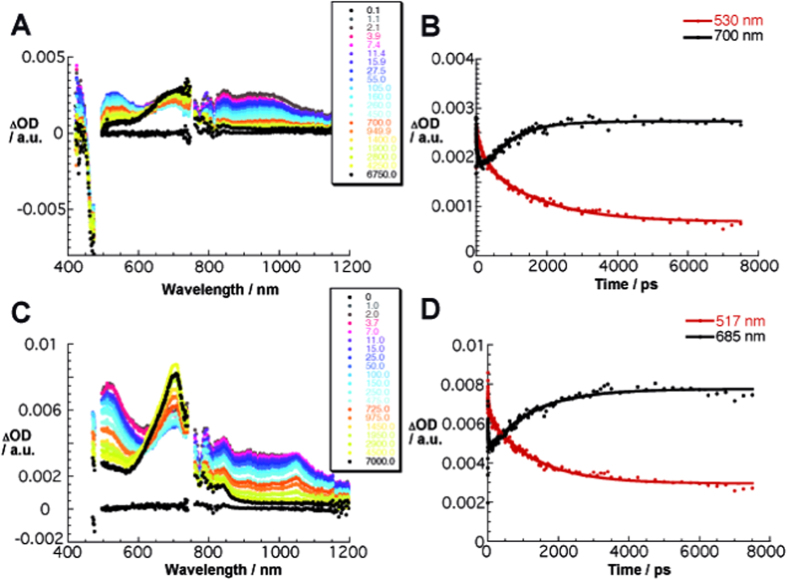
Differential absorption spectra for the n/p-nanohybrids. (**A**) Differential absorption spectra (visible and near-infrared) obtained upon femtosecond flash photolysis (480 nm) of **1**:C_60_ in TCE with several time delays between 0.1 and 6750.0 ps at room temperature. (**B**) Time-absorption profiles of the spectra at 530 and 700 nm, monitoring the charge separation/charge recombination. (**C**) Differential absorption spectra (visible and near-infrared) obtained upon femtosecond flash photolysis (480 nm) of **1**:PCBM in TCE with several time delays between 0 and 7000.0 ps at room temperature. (**D**) Time-absorption profiles of the spectra at 517 and 685 nm, monitoring the charge separation/charge recombination.

Considering the composition of the n/p-nanohybrid, that is, an electron donating exTTF and an electron accepting C_60_, we turned to pump probe experiments (Fig. 4). In reference experiments, exTTF, C_60_, and PCBM were probed in solution-based experiments. To this end, exTTF reveals upon radiation at 480 nm a short lived excited state spectrum with features that include transient maxima at 695 nm. From multi-wavelength analyses a lifetime of 0.075 ± 0.015 ns was determined, by which the photoexcited exTTF decays to the singlet ground state. An efficient second order spin coupling is responsible for this fast process. In stark contrast, for C_60_/PCBM a much slower deactivation – on the order of 1.5 ± 0.2 ns – of the singlet excited state transients is noted. Products are, however, the triplet excited states, which are formed in any of the cases nearly quantitatively. The most notable transient features of the singlet and triplet excited states are for C_60_ maxima at 965 and 750 nm, respectively. For PCBM, these maxima are shifted towards 960 and 690 nm.

Pump probe experiments with the n/p-nanohybrid let immediately upon photoexcitation to differential absorption spectra, which are sound in agreement with the features of the C_60_ singlet excited state. These decay, however, in contrast to what has been seen in the reference experiments on the time scale of less than 100 ps and transform into a new species. The latter resembles with transient maxima at 700 and 1030 nm those seen upon the one electron oxidation of exTTF and the one electron reduction of PCBM, respectively. In the case of C_60_, the near-infrared maximum evolved at around 1080 nm. As such, we conclude that photoexcitation of the self-assembled nanohybrids proceeds via a transient excited state into a charge separated state. A kinetic analysis across the visible and near-infrared assisted in gathering the rate constants for charge separation and charge recombination. In particular, for C_60_ and PCBM the values were 2.6 × 10^10^/1.1 × 10^9^, 1.2 × 10^11^/8.1 × 10^8^ s^−1^, respectively. Interesting is that in these cases only one long lived transient is discernable after the conclusion of the charge recombination, namely the triplet excited state of either C_60_ or its derivatives.

**Figure 5:**
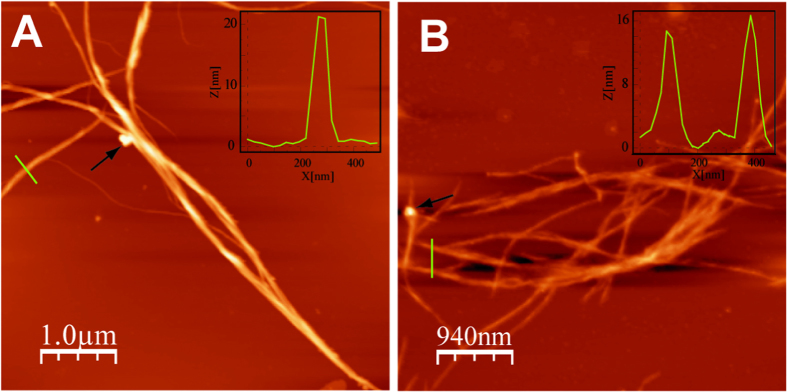
Atomic Force Microscopy (AFM) images and height profiles of the n/p-nanohybrids. AFM images and the corresponding height profiles of the nanohybrids (**A**) **1**:C_60_ (2.6 × 10^−4^ M) and (**B**) **1**:PCBM (2.6 × 10^−4^ M) showing the presence of nanohybrid fibers with an increase in height with respect to the initial exTTF-fibers. Arrows indicate C_60_ or PCBM domains over the fibers.

AFM and TEM microscopic images provide valuable information not only about the nanohybrid’s morphology, but also about the growth of crystalline nuclei on the surface of the nanohybrids. To this end, a TCE solution containing exTTF-fibers and C_60_ was left at room temperature for 5 days. Afterwards, the mixture was drop-casted onto mica surfaces and air-dried. The AFM images revealed the presence of more straight fibers (Fig. 5A) relative to the initial exTTF-fibers^50^. AFM height profiles showed that the new fibers are higher (14–22 nm) than those obtained from only **1** (2–10 nm) (see insets in Fig. 5). Such an increase in size, points to the nanohybrid formation (Fig. 5A). A similar behavior was observed for the mixtures of exTTF-fibers and PCBM (Fig. 5B). Again, notable changes with respect to the height values and the morphology confirmed the integration of PCBM into the exTTF-fibers (Fig. 1A)^51^,^52^,^53^,^54^. From Fig. 5 we discern crystals of C_60_ (see arrow in Fig. 5A) and PCBM (see arrow in Fig. 5B), indicating the selective crystallization guided by the nanohybrid and triggered by the local saturation and high tendency of C_60_ and PCBM to aggregate (Fig. 1B).

**Figure 6:**
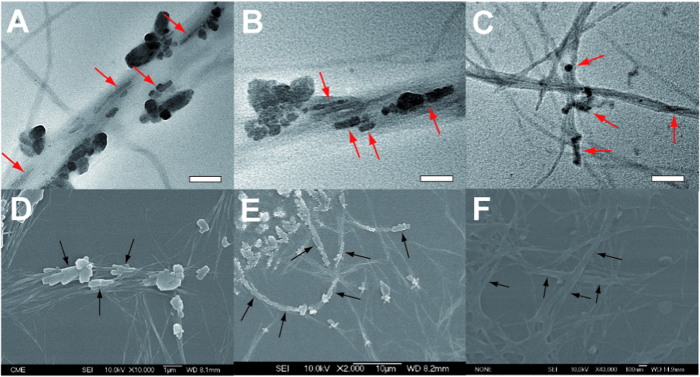
Transmission electron microscopy (TEM) and scanning electron microscopy (SEM) for the n/p-nanohybrids. (**A**–**C**) are TEM images of both nanohybrids (**1**:C_60_, 1.5 × 10^−4^ M, (**A,B**) and (**1**:PCBM, 1.5 × 10^−4^ M, (**C**), arrows remark the presence of C_60_ or PCBM domains along the fiber’s axis. Scale bars: 200 nm. (**D**–**F**) SEM images of both nanohybrids (**1**:C_60_, 4.3 × 10^−4^ M, (**D,E**) and (**1**:PCBM, 4.3 × 10^−4^ M, F), arrows show the lengthening of C_60_ and PCBM domains grown in the axial direction of the fibers.

The aforementioned AFM observations were further supported by TEM experiments carried out for both nanohybrids. In the case of C_60_, TEM images showed the presence of large straight fibers, in which C_60_ crystal nuclei are discernable (Fig. 6A–B, arrows). In a similar way to that observed in biomimetic mineralization processes, the crystals grow along the fiber’s axis^35^. To shed light on the formation of C_60_ crystals over the fibers, HRTEM measurements were performed. It is clearly distinguished C_60_ crystal lattices with a distance around 1.1 nm which is in aggrement with the PXRD results (see Supplementary Fig. 4). Also SEM experiments corroborate the oriented C_60_ crystal growth exclusively on fiber’s surfaces (see arrows in Fig. 6D–E). Futhermore this tendency was also observed for PCBM based nanohybrid, where PCBM domains grow along the fiber’s axis (Fig. 6C,F).

In control experiments, the presence of exTTFs in the nanofibers was probed towards the oriented C_60_/PCBM crystal growth. As a matter of fact, fibers featuring the same pentapeptide sequence (Ala-Gly-Ala-Gly-Ala-NH_2_) linked, to a π-aromatic fluorene unit (**2**, for chemical structure see supplementary information Fig. 4) were employed^45^. Initially, we fabricated fluorene-nanofibers from TCE solutions of **2** and added C_60_. The mixture was aged for 5 days before they were characterized by SEM (see Supplementary Fig. 5). In this particular case, most C_60_ crystals evolved in domains that lack any fiber. This fact confirms the crucial role of exTTF in the nanofibers in terms of inducing crystallization of C_60_/PCBM.

## Conclusions

In summary, the above described results point to the successful formation of 1D n/p-nanohybrids based on the efficient interfacing of exTTF-fibers with C_60_/PCBM, where electron donor and electron acceptor are nanostructured at the same length scale. The efficient coating of exTTF-fibers by C_60_/PCBM promotes the guided crystallization of C_60_/PCBM on top of exTTF-nanofibers. In addition, fast charge separation and slow charge recombination in 1D n/p-nanohybrids afforded long lived transients, which attests electronic interactions between the electroactive species upon light irradiation. The results presented here contribute to the progress in the development of easy and general proceedings for building up 1D n/p-nanostructures, in which a better organization between electron donors and electron acceptors is achieved – a feature, which is highly demanded in the controlled construction of new efficient photo-active materials.

## Methods

### General methods

The synthesis of compound **1** was performed according to reference 50. ^1^H-NMR and ^13^C-NMR spectra were recorded on a Bruker Avance (700 MHz for ^1^H-NMR and 125 MHz for ^13^C-NMR). Unless otherwise indicated, chemical shifts (δ) are reported in ppm downfield from tetramethylsilane (TMS) at room temperature using deuterated solvents as internal standard. UV-Vis spectra were recorded in a UV-3600 Shimadzu Spectrophotometer. Circular Dichroism spectra were performed in a Jasco *J*-815 CD spectrometer at room temperature. Mass spectra by electrospray ionization (ESI) were recorded on a HP1100MSD spectrometer. Column chromatography was carried out on Merck silica gel 60 (70–230 mesh). Reagents were purchased from Sigma-Aldrich and were used without further purification. FTIR spectra were recorded on a Bruker Tensor 27 (ATR device) spectrometer. Raman spectra were acquired with a Renishaw inVia confocal Raman microscopy instrument, equipped with 532 nm on a glass microscope slide.

### Powder X-ray Diffraction (PXRD)

X-ray diffraction was performed in a Panalytical X’Pert PRO diffractometer with Cu tube (lambda Kα = 1.54187 Å) operated at 45 kV, 40 mA, Ni beta filter, programmable divergence and anti-scatter slits working in fixed mode, and fast linear detector (X’Celerator) working in scanning mode. In the case of exTTF-fibers (**1**), the PXRD measurements were obtained from a dispersion of **1**, which was prepared by dropping over toluene an initial solution of **1** in TCE. This dispersión was drop-casted over a silica support and air dried at room temperature for 24 h.

### Transmission Electron Microscopy (TEM)

TEM images were performed using a JEOL 2000-FX electron microscope, operating at 200 kV accelerating voltage. The samples were prepared by drop-casting the respective n/p-nanohybrids (1.5 × 10^−4^ M) on a carbon film 200 square mesh copper grid (CF200-Cu). TEM images were obtained after drying the sample by air for 24 hours.

### Atomic Force Microscopy (AFM)

AFM images of the different nanohybrids were acquired under ambient conditions using SPM Nanoscope IIIa multimode working on tapping mode with a RTESPA tip (Veeco) at a working frequency of ~235 Khz. The samples were prepared by drop-casting of the respective n/p-nanhybrids solutions (2.6 × 10^−4^ M) on a freshly cleaved mica surface and were dried under ambient conditions for 24 hours.

### Scanning Electron Microspopy (SEM)

SEM images were acquired by using a JEOL JSM 6335F microscope working at 10 kV. The nanohybrids (4.3 × 10^−4^ M) were deposited by drop-casting on a silicon substrate, dried under ambient conditions and metallized with Au before observation.

### Acquisition of Transient Absorption Spectroscopy

Differential transient absorption studies were performed with an amplified Ti/sapphire laser system (Model CPA Hybrid 2110, Clark-Max Inc., output: 775 nm, 1 kHz, and 150 fs pulse width). The 480 nm excitation pulses (200 nJ energy) were used as pump pulses and white light continuum as probe beam. The samples were placed in quartz cuvettes with 2 mm path length. Data aqusion was performed employing commercially-built spectrometer (Helios from Ultrafast systems).

### General method for 1:C_60_ (or PCBM) nanohybrid preparation

The nanohybrids were prepared as follows: To a solution of compound **1** (1 mg/1 mL) in 1,1,2,2-tetrachloroethane (TCE) was added one equivalent of C_60_ (or PCBM). In the case of C_60_, sonication for 5 minutes was performed in order to solubilize C_60_ completely. In the case of PCBM no sonication was required. These mixtures were aged at least for 5 days before exploring by microscopic techniques.

## Additional Information

**How to cite this article**: Insuasty, A. *et al.* Supramolecular One-Dimensional n/p-Nanofibers. *Sci. Rep.* **5**, 14154; doi: 10.1038/srep14154 (2015).

## Supplementary Material

Supplementary Information
