# Supplementary material for: Supramolecular One-Dimensional n/p-Nanofibers
Source: Sci Rep. 2015 Sep 15;5:14154. doi: 10.1038/srep14154 (PMC5378913; doi:10.1038/srep14154)
Supplement: Supplementary Information [file srep14154-s1.pdf]

Supramolecular One-Dimensional n/p-Nanofibers

Alberto Insuasty,[a] Carmen Atienza,[a] Juan Luis López,[a] Juan Marco-Martínez,[a] Santiago Casado,[b] Avishek Saha,[c] Dirk M. Guldi[c*] and Nazario Martín[a,b*]

[a]Departamento de Química Orgánica I, Facultad de Ciencias Químicas, Universidad Complutense, E-28040 Madrid, Spain

[b]IMDEA- Nanoscience, Campus de Cantoblanco, E-28049 Madrid, Spain

[c]Department of Chemistry and Pharmacy & Interdisciplinary Center for Molecular Materials, Friedrich-Alexander-University Erlangen-Nuremberg, 91058 Erlangen, Germany

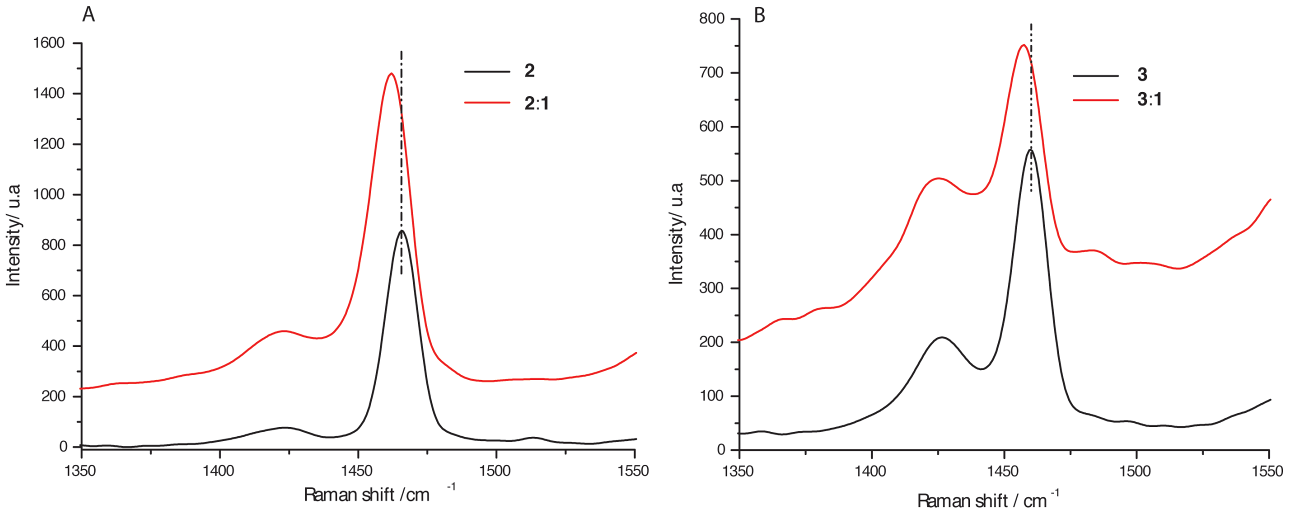

Supplementary Figure S1. Raman spectra of pristine C_{60}, PCBM and nanohybrids. Raman spectra of C_{60} and PCBM as references (black spectrum) and n/p-nanohybrids **1**:C_{60} (A) and **1**:PCBM (B) (red spectrum) on glass microscope slide - excitation wavelength 532 nm. For the nanohybrids (**1**:C_{60} and **1**: PCBM) a shift from the respective reference is observed, this observation together with the changes that were observed in other spectroscopic techniques (UV-Vis and CD) support the formation of the n/p-nanohybrids.

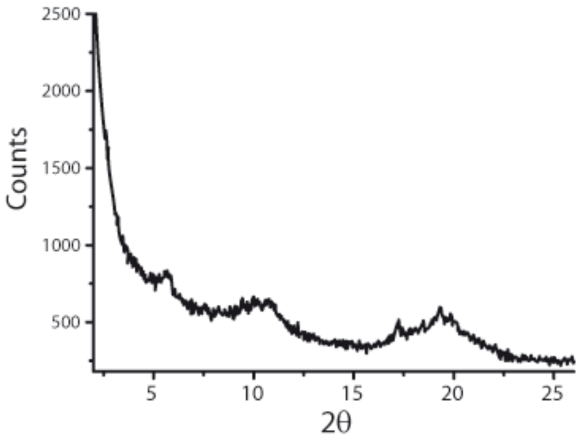

Supplementary Figure S2: Powder-XRD experiment carried out for nanohybrid **1:**PCBM in TCE.

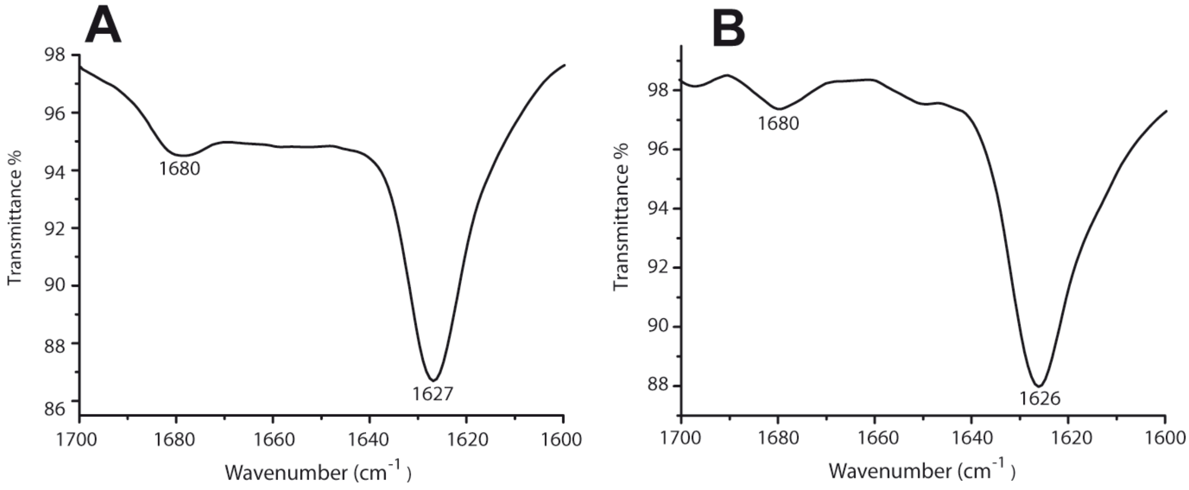

Supplementary Figure S3: ATR-FTIR of n/p-nanohybrids **1**:C_{60} (A) and **1**:PCBM (B), respectively. Both IR spectra show the amide I band at 1627 cm^{-1} and a weak shoulder around 1680 cm^{-1} which means that the β-sheet is retained within the nanohybrid nanostructure.

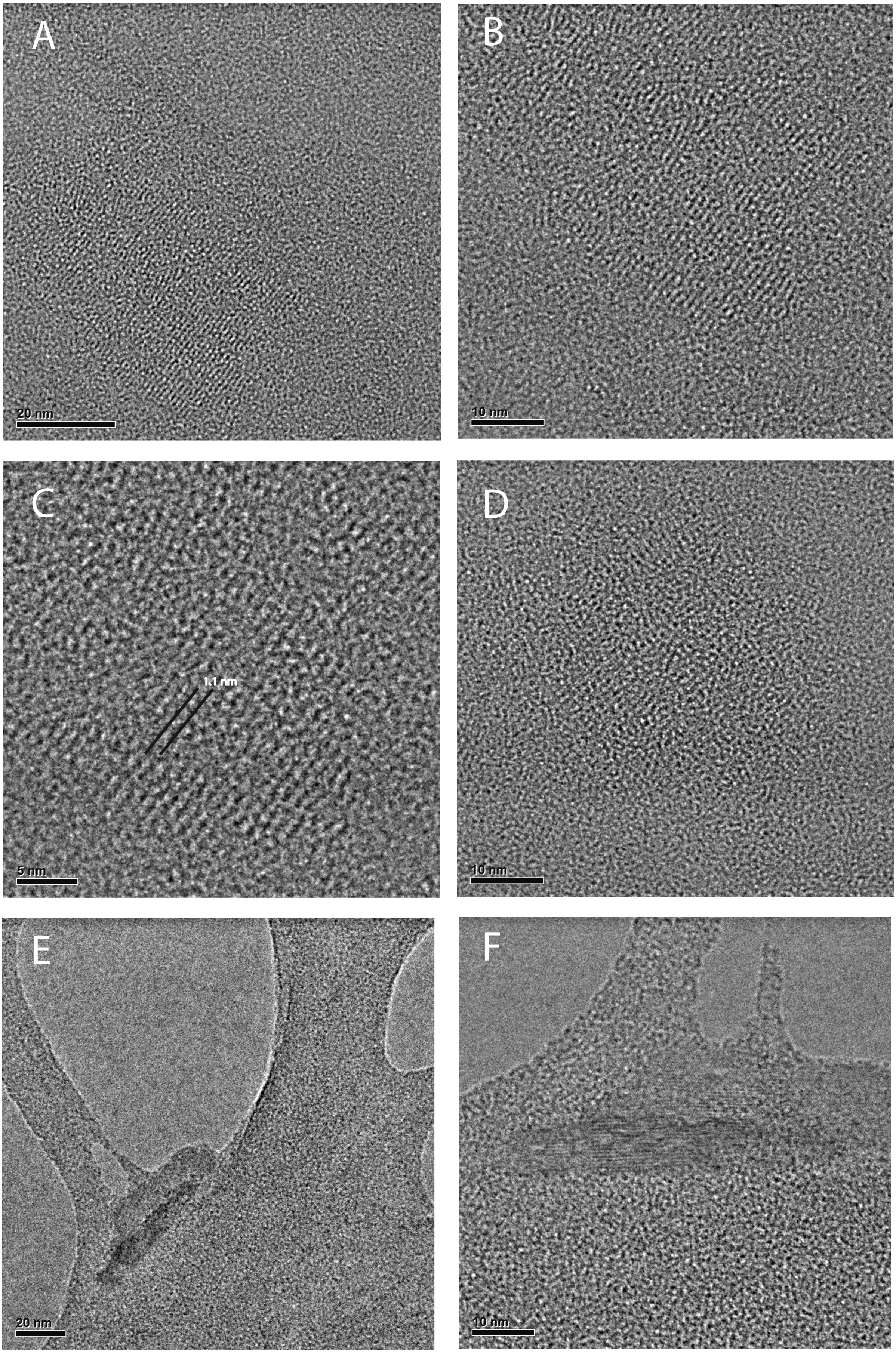

Supplementary Figure S4: A-F) HRTEM images obtained from the nanohybrid **1**:C_{60}

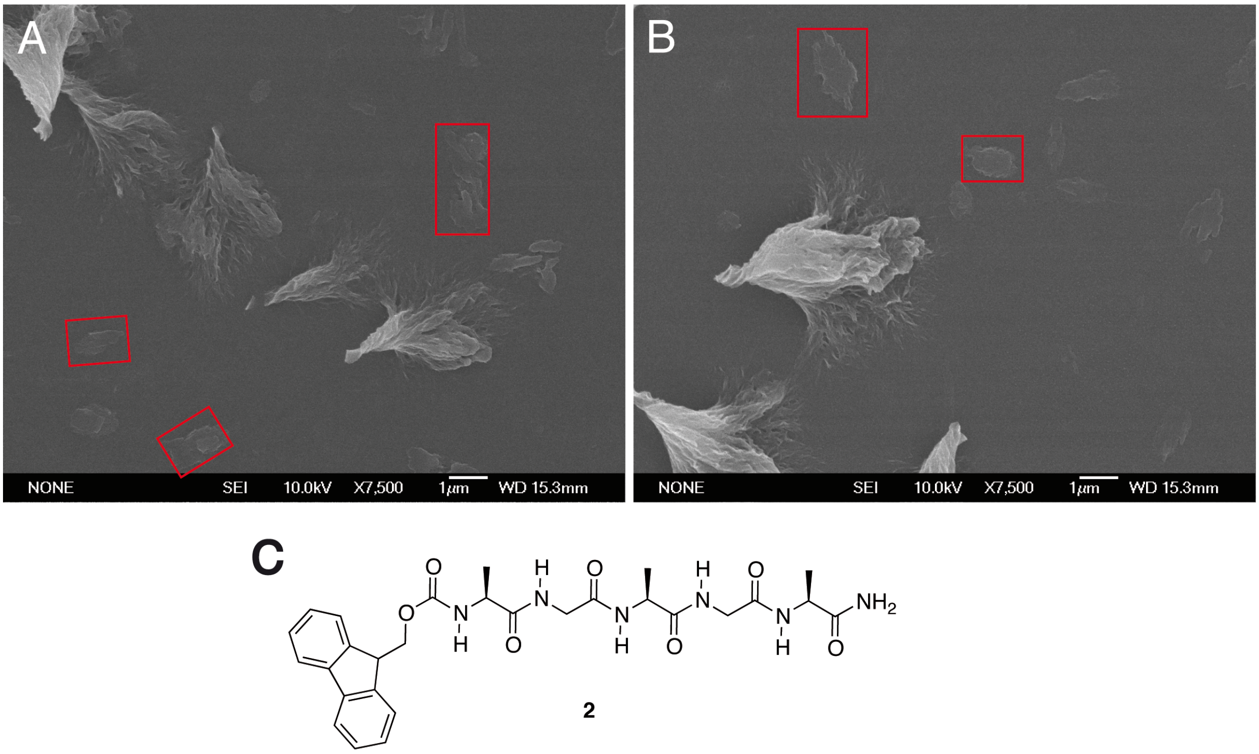

Supplementary Figure S5: A) and B) SEM images obtained from the mixture of **2**:C_{60} aged for 5 days. It is clearly observed that most C_{60} crystals evolved in domains that lack any fiber. C) Chemical structure of reference compound (**2**) based on the same pentapeptide sequence used for **1** covalently linked to a fluorene moiety.
